# Reduced ectoparasite load, body mass and blood haemolysis in Eurasian kestrels (*Falco tinnunculus*) along an urban–rural gradient

**DOI:** 10.1007/s00114-021-01745-x

**Published:** 2021-09-07

**Authors:** Laura Wemer, Arne Hegemann, Caroline Isaksson, Carina Nebel, Sonia Kleindorfer, Anita Gamauf, Marius Adrion, Petra Sumasgutner

**Affiliations:** 1 Department of Integrative Zoology, University of Vienna, Vienna, Austria; 2 Natural History Museum Vienna, Vienna, Austria; 3 Department of Biology, Lund University, Lund, Sweden; 4 FitzPatrick Institute of African Ornithology, DSI-NRF Centre of Excellence, University of Cape Town, Cape Town, South Africa; 5 NABU E.V, Head office, Berlin, Germany; 6 Konrad Lorenz Research Centre, Core Facility for Behaviour and Cognition, Department of Behavioral & Cognitive Biology, University of Vienna, Vienna, Austria; 7 College of Science and Engineering, Flinders University, Bedford Park, SA 5001 Australia; 8 Department of Biology, University of Turku, Turku, Finland

**Keywords:** Glutathione, Haptoglobin, Body condition, Oxidative stress, Ectoparasite, Falconiformes

## Abstract

Urbanisation is proceeding at an alarming rate which forces wildlife to either retreat from urban areas or cope with novel stressors linked to human presence and activities. For example, urban stressors like anthropogenic noise, artificial light at night and chemical pollution can have severe impacts on the physiology of wildlife (and humans), in particular the immune system and antioxidant defences. These physiological systems are important to combat and reduce the severity of parasitic infections, which are common among wild animals. One question that then arises is whether urban-dwelling animals, whose immune and antioxidant system are already challenged by the urban stressors, are more susceptible to parasitic infections. To assess this, we studied nestlings of Eurasian kestrels (*Falco tinnunculus*) in Vienna, Austria, during 2015 and 2017. We measured biomarkers of innate immune function, oxidative stress and body mass index and ectoparasite infection intensity in 143 nestlings (from 56 nests) along an urban gradient. Nestlings in more urbanised areas had overall fewer ectoparasites, lower haemolysis (complement activity) and lower body mass index compared to nestlings in less urbanised areas. None of the other immune or oxidative stress markers were associated with the urban gradient. Despite some non-significant results, our data still suggest that kestrel nestlings experience some level of reduced physiological health, perhaps as a consequence of exposure to more urban stressors or altered prey availability in inner-city districts even though they had an overall lower ectoparasite burden in these heavily urbanised areas.

**Supplementary Information:**

The online version contains supplementary material available at 10.1007/s00114-021-01745-x.

## Introduction

Urbanisation is rapidly transforming natural environments into altered landscapes with measurable effects on health and fitness of humans and wildlife (McKinney 2008; McDonnell & Pickett 1990; Kawecki and Ebert, 2004). Animals living in urban environments encounter physical changes (sealed surfaces) and a range of so-called urban stressors (i.e. urban factors that can be challenging and even stressful) such as exposure to anthropogenic activity and noise (Slabbekoorn and Ripmeester, 2008), artificial light at night (Longcore & Rich 2004; Falchi et al. 2016; Gaston et al. 2015) and/or chemical pollution (Vitousek et al. 1997). In addition, many urban dwellers experience conditions of lower food quality linked to anthropogenic ‘junk’ food (Stofberg et al. 2019) and lower availability of natural food (Sumasgutner et al. 2014a; Risi et al. 2021). Furthermore, they are often exposed to and infected by different parasite and pathogen assemblages compared to non-urban conspecifics (Dhondt et al. 2005; Giraudeau et al. 2014). As a consequence, urban-dwelling animals show pronounced behavioural and physiological differences compared to their rural conspecifics. For example, urban birds can have altered biological clocks (Helm et al. 2013), paler carotenoid-based coloration (Isaksson et al. 2005; Sumasgutner et al. 2018), increased oxidative stress (Isaksson et al. 2005; Costantini et al. 2014; Isaksson, 2010, 2015; Herrera-Dueñas et al., 2014, 2017) and lower immune competence (Chávez-Zichinelli et al. 2013). All these factors can negatively impact their health, reproductive performance and survival (Isaksson, 2015; Salmón et al. 2017; reviewed by Chamberlain et al. 2009).

The redox and innate immune system are of fundamental importance for all animals, and they are particularly relevant for coping with environmental challenges (Isaksson 2015). These two systems are highly inter-correlated (Cotran et al., 1995; Halliwell & Gutteridge 2002). Yet, most studies have mainly focused on a single physiological system and, as a result, lack an integrative perspective (but see Ibáñez-Álamo et al. 2020). The redox system is driven by antioxidants that are an important resource for detoxifying pro-oxidative pollution such as nitrogen oxides (NO_x_), particulate matter and heavy metals. Antioxidants can either be endogenously synthesized (e.g. glutathione (GSH) and catalyse) or dietary (e.g. carotenoids and α-tocopherol); thus, both intrinsic capacities and dietary/habitat quality can influence the overall antioxidant defences. A prolonged or a too high exposure to urban pro-oxidative air pollution can lead to oxidative stress and thereby increased oxidative damage to proteins, lipids and DNA, with negative effects on cellular function followed by various negative health effects (Liu et al. 2004; Yeh et al. 2006; Isaksson 2010; Chávez-Zichinelli et al. 2013). Although urban birds suffer from increased exposure to these pro-oxidative pollutants, their antioxidant responses show mixed consequences (i.e. linked to for example disease status, life history, diet or degree of urbanisation: Costantini et al. 2014; Herrera-Dueñas et al. 2014; Isaksson 2020). The paradox with the redox system is that the antioxidant system should not be too efficient in its action towards pro-oxidants. For example, different immune cell types such as phagocytes and neutrophils can produce and purposely release pro-oxidants (often referred to as reactive oxygen species, ROS) to attack and destroy pathogens in the body, which brings the close interaction with the immune system.

An increased oxidative stress has also been revealed in response to ectoparasites, with implications for carotenoid-based signals (Mougeot et al. 2010) and experimental evidence showed that ectoparasites reduce the antioxidant defence in nestlings of pied flycatchers *Ficedula hypoleuca* (López-Arrabé et al. 2015). There are correlative data suggesting that individuals with less carotenoid-based colouration in Eurasian kestrels *Falco tinnunculus* in more urbanised areas had more ectoparasites, which is supposedly consistent with investment of dietary carotenoids into the immune system (Sumasgutner et al. 2018). Paler plumage colouration has also been found in urban great tits (*Parus major*) and was related to elevated oxidative stress (Isaksson et al. 2005) and measures of pollution (Eeva et al. 1998). While this highlights the need for proper nutrition and dietary antioxidants, such as carotenoids, to mitigate oxidative stress during an infection, experimental evidence thus far could not confirm that carotenoids used in the immune system are traded-off against plumage coloration when exposed to oxidative stress (Isaksson and Andersson 2008; Stirnemann et al. 2009).

Although the inflammatory release of ROS is an effective first line of defence against pathogens, it is non-targeted, thereby also posing oxidative harm to the individual. To limit further oxidative damage, ROS simultaneously regulates and induces other parts of the innate immune system; for example, the ability of plasma to agglutinate and lyse antigens (Matson et al. 2005), mainly driven by natural antibodies and the complement system. Another mechanism includes release of haptoglobin, an acute-phase protein that is released from the liver during infection (Abbas et al. 2012). Similar to the redox system, inflammatory markers are activated when exposed to pollution such as NO_x_ or particulate matter (Glencross et al. 2020). Thus, to study parts of these two systems simultaneously will provide a better systemic overview of the health of urban dwellers.

To function well, both the redox system and the immune system need a well-nourished state with respect to energy and specific nutrients (Klasing, 2004). This can be an additional challenge for urban-dwellers (Plummer et al. 2019). The urban diet composition can vary by food source, prey type and/or quality (Isaksson 2015; Stofberg et al. 2019), with evidence that some urban pollutants can accumulate at higher trophic levels (Drouillard et al. 2001; Henny et al. 2003; Ortiz-Santaliestra et al. 2015). Urban habitats can be attractive for some raptors because buildings provide suitable nesting sites for many cavity nesters. Yet, if those species rely on small mammals as prey, food availability might be lower than in non-urban settings (Sumasgutner et al. 2014b; Kettel et al. 2018), with negative impacts on immune function and redox system. These combined effects could therefore enhance the negative effects of oxidative stress, inflammation and susceptibility to parasites in raptors.

Eurasian kestrels are urban raptors (Cramp & Tomlins 1966; Kostrzewa & Kostrzewa 1993; Kübler et al. 2005) that are likely attracted to inner-city districts by abundant nesting opportunities (Village 1983; Sumasgutner et al. 2014b). However, the high percentage of sealed surface areas correlates with a low availability of diurnal voles (Sumasgutner et al. 2014a; Mitter et al. 2015), which form their main prey under natural conditions (Village 1990). Thus, urban kestrels are either forced to extend their foraging trips to rural hunting grounds (Riegert et al. 2007) or to enrich their diet with alternative prey available in the centre, especially passerines, but also lizards and insects (Kübler et al. 2005; Düesberg 2012; Sumasgutner et al. 2013, 2014b; Kreiderits et al. 2016). This diet alteration can increase starvation-related mortality in urban nestlings, resulting in lower breeding success of urban pairs (Sumasgutner et al. 2013, 2014a, b). While adult kestrels are highly mobile and thus not necessarily exposed to urban stressors around the clock, their nestlings must endure variation in food availability, sibling competition and exposure to parasites and predators, as well as adverse weather and other environmental stressors. At the same time, some of these stressors might be buffered by parental care and sheltering effects of cavities (Blas et al. 2005; Sumasgutner et al. 2020). Furthermore, during this period of crucial structural growth, several physiological systems are still in development, including innate immune function (Aastrup & Hegemann 2021).

In this study, we investigate the effects of urbanisation on biomarkers of oxidative stress, innate immune function, body mass and ectoparasite infection in nestlings of Eurasian kestrels along an urbanisation gradient based on sealed surfaces. As oxidative stress markers, we measured glutathione (GSH) and its oxidised form (glutathione disulphide, GSSG). GSH is considered the most important intra-cellular antioxidant and detoxifier (Kaplowitz 1981) as it scavenges endogenously produced or inhaled pro-oxidants and conjugates industrial toxins. When GSH is used as a scavenger, the GSH is oxidised to GSSG and the ratio between GSH and GSSG is commonly used as a biomarker of oxidative stress (Kidd 1997; Pompella et al. 2003; Townsend et al. 2003; van der Oost et al. 2003). Therefore, we predict a lower GSH:GSSG ratio with increased urbanisation and ectoparasite intensity. In addition, total level of glutathione (tGSH, the sum of GSH and GSSG) is a biomarker of toxin exposure (reviewed in Isaksson 2020), which is predicted to be lower in response to increased urbanisation. As markers for innate immune function, we measured complement (lysis titres), natural antibodies and haptoglobin concentrations. Combining these immune markers allows for a more comprehensive view on innate immune function (Boughton et al. 2011; Demas et al. 2011; Salvante 2006; Hegemann et al., 2017). In response to urbanisation, we predict that nestlings in poorer nutritional state will have reduced levels of complement activity and natural antibodies, while haptoglobin concentrations might correlate positively with ectoparasite infections.

## Material and methods

### Study species and study area

The Eurasian kestrel, hereafter ‘kestrel’, is a common raptor in Europe, Asia and Africa (Village 1990). This study was done in the city of Vienna, Austria (48° 12′ N, 16° 22′ E; 415 km^2^, 1.88 million inhabitants), where 350 to 400 Eurasian kestrel pairs breed (Sumasgutner et al., 2014a, b). We sampled kestrel nestlings along an urbanisation gradient defined by the percentage of sealed surface (see below), because this has been shown to be the most important predictor variable for kestrel prey type (Kübler et al 2005; Rejt 2001; Salvati et al 1999). We expected the primary urbanisation impact to arise from changes in food availability and diet composition because of the strong relationship between sealed surface and abundance of the main prey items (Kübler et al. 2005; Sumasgutner et al. 2014a, b). The total study area was 243 km^2^, excluding areas of less than 1% sealed surface, i.e. the Viennese Forest in the West, which is unsuitable habitat for the species, and vast agricultural areas in the East, where our monitoring capacity was limited. The degree of urbanisation was calculated as the percentage of sealed surface within a 500-m radius around each nesting site, using ArcGIS 10 by ESRI©, based on land covered by buildings and traffic areas (see Sumasgutner et al. 2014a for details).

Until now, many studies rely on a simplified urban–rural comparison with two study sites or a limited number of urban–rural replicates, which do not necessarily vary in their degree of urbanisation alone but likely also in local weather parameters or biotic interactions that go beyond urban–rural differences. Applying a gradient approach allows a higher resolution of environmental variables and conclusions that are more likely linked to urbanisation per se. Along with a higher proportion of sealed surface areas, human population density (Stankowski 1972) intensity of chemical pollution (Andrews 2008; Krommer et al. 2007; Mingorance & Oliva 2006; Simon et al. 2011), noise pollution (Mendes et al. 2011; Pijanowski et al. 2011), artificial light pollution (Cinzano et al., 2001, 2007; Hölker et al. 2010) and infectious diseases (Bradley & Altizer 2007; Giraudeau et al., 2014) are usually increasing along urbanisation gradients. Thus, there might be impacts from multiple origins (Isaksson 2015; Andrews 2008; Mendes et al. 2011; Cinzano et al. 2007).

### Morphometric measurements, blood sampling and ectoparasite count

**Fig. 1:**
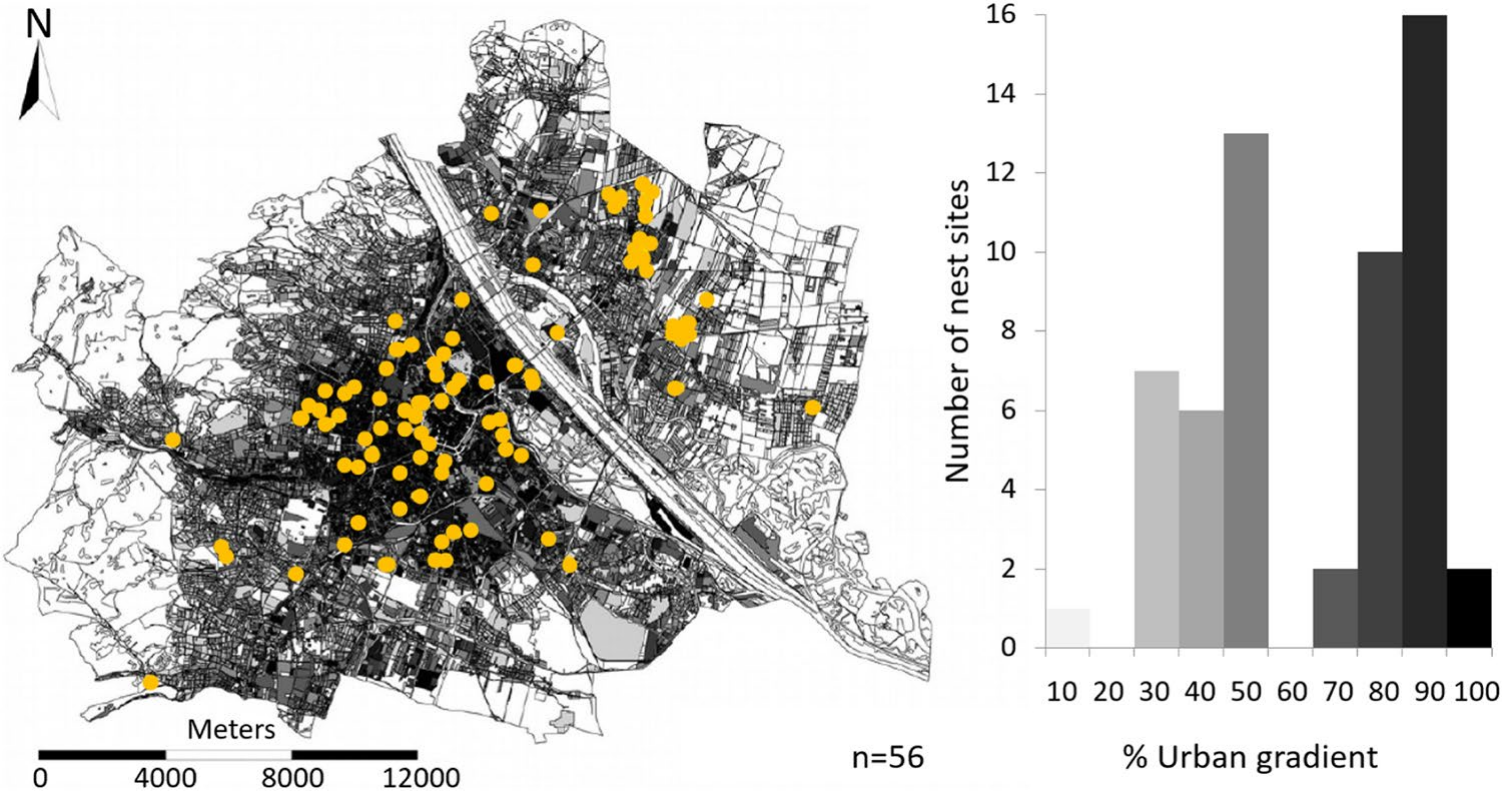
Urban study area (243 km^2^) in Vienna, Austria. The urban gradient is displayed from light grey to black (white areas (< 1%) are largely forested and therefore unsuitable habitat for kestrels and not monitored); (left) locations of Eurasian kestrel *Falco tinnunculus* nest sites are displayed in yellow (*n*_2015_ = 30; *n*_2017_ = 26); and (right) distribution of nest sites displayed in ten categories (*n* = 56 nests)

Kestrel nesting sites were located by visual observation during the breeding season, from February to July, and were regularly checked for occupancy by researchers and volunteers of the Viennese Kestrel Project (see Huchler et al. 2020 for details). We used data from 56 different nesting sites that were visited three to six times during the breeding season of either 2015 or 2017 (Fig. 1). The following breeding parameters were determined: laying date (relevant for age estimation) and brood size (total number of hatched offspring). Laying date is defined as the day the first egg was laid and was either observed directly or back-dated based on the nestlings’ plumage development and wing length during ringing (for the method see Kostrzewa & Kostrzewa 1993 and Birdlife Finland, http://netti.nic.fi/). As both methods are based on European kestrel populations outside our study area (Germany and Finland), we used the average of both values (see Sumasgutner et al. 2018) to determine the hatching rank within the brood (Martinez-Padilla & Viñuela 2011; Hardey et al. 2013; reviewed in Amundsen & Slagsvold 1996): the siblings were denoted as either senior (‘1’, first-hatched/largest), junior (‘3’, last-hatched/smallest) or intermediate (‘2’, all the other nestlings in between; brood size up to 6).

At the age of approximately 12 to 16 days, each nestling (*n* = 195 from 56 broods) was ringed with a metal ring from the local ringing centre and one individually engraved colour ring (Ecotone, Poland). Body morphometrics were taken by measuring body mass (g) and wing length (to the nearest mm, Eck et al. 2011). To calculate a nestling’s body mass index (BMI), we followed the method described by Roulin et al. (2007): We extracted the residuals of a linear model with body mass as the response and wing length (linear and quadratic) and sex (males being 20% smaller than females in body mass, Village 1990) as explanatory variables.

We collected approx. 200 µl of blood by puncturing the brachial vein of each nestling. For the glutathione assay, 10 µl of full blood was immediately frozen in liquid nitrogen to prevent oxidation of GSH to GSSG and later stored at − 80 °C. Remaining blood was placed on ice and centrifuged (10,000 rpm for 10 min) within 4 h of sampling to separate plasma and red blood cells and then stored at − 15 °C. Due to varying field protocols between years, full blood samples for the GSH and GSSG (*n* = 143) were collected in 2015 and 2017, whereas blood plasma for the immune assays were collected only in 2017 (*n* = 69 nestlings from 26 broods). Sample sizes among assays varied slightly due to sample volume limitations and can be found in Appendix C for respective assays. Red blood cells were used for molecular sex determination (Fridolfsson & Ellegren 1999).

All 195 nestlings were screened for ectoparasites (most abundant arthropod parasite: *Carnus hemapterus*) by counting all parasites on the surface of both wings and the rump without removing them in the process. Ectoparasite infection intensity was classified based on these counts directly in the field as follows: 0 = no ectoparasites, 1 = one to three ectoparasites, 2 = three to nine ectoparasites; 3 = 10 or more parasites (see Sumasgutner et al. 2018 for a similar approach). We did not consider other ectoparasites as we only occasionally encountered ticks and louse flies on the nestlings while screening. Hence, sample sizes of other ectoparasites were not sufficient for analysis.

### Lab procedure

All samples were randomized before lab work and were analysed blind with respect to degree of urbanisation or timing of sampling (i.e. early and late broods). Full details for measurements of total glutathione (tGSH) and GSSG (full blood) can be found in the supplementary material. To quantify haptoglobin concentrations, a commonly used marker for inflammation levels (Abbas et al. 2012), we used a commercially available colorimetric assay kit (TP801 Kit, Tri-Delta Diagnostics, Ireland) (Matson et al 2006). To quantify titres of complement-like lytic enzymes (lysis titre) and nonspecific natural antibodies (agglutination titre), we used a haemolysis-haemagglutination assay (Matson et al. 2005). The detailed laboratory protocols can be found in the supplementary material.

### Statistical analysis

The biomarkers for oxidative stress (GSH:GSSG ratio and tGSH), immune parameters (haptoglobin, haemagglutination and haemolysis) and BMI were each used as response variables in multiple linear mixed models (LMMs) with urban gradient (in %) as the key explanatory covariate. Response variables followed a Gaussian distribution and were fitted with either an identity-link function or a logit transformation (haptoglobin). To control for additional factors that could influence physiology and the BMI, we fitted ectoparasite infection intensity, brood size, sex and hatching rank of the nestlings to the model (after checking for potential correlation of fixed effects). We did not further control for nestling’s age (in days) as it was (i) strongly correlated with hatching rank; and (ii) hatching rank was stronger in explanatory capacity than age, and seemed biologically more meaningful. All continuous variables were scaled and centred (standardised to mean of 0 and SD = 1) to bring the variables to comparable dimensions across years (Schielzeth 2010), sex and plate ID (where applicable, see below) were defined as factor variables and ectoparasite infection intensity and hatching rank as ordered factor variables. Results in the model output are thus displayed for the linear, quadratic or cubic relationship between the different levels of parasite infection intensity, and linear or quadratic relationship for hatching rank between junior, middle and senior sibling. The variables tGSH and GSH:GSSG ratio were additionally standardised within each year to account for possible year effects and different storage times.

In the ‘oxidative stress models’ (GSH:GSSG, tGSH), the plate ID was added as an additional predictor variable to account for the potential plate effect from laboratory work. In the ‘haptoglobin model’, the additional variable was a reading at 405 nm to control for plasma colouration (Matson et al. 2012) and in the ‘body mass index model’, the corresponding GSH:GSSG ratio was added to explore a potential influence. We did not include any of the immune assays in this analysis, as we had a limited sample size. In all models, we included the brood ID as a random factor to control for pseudoreplication (Hurlbert 1984) because siblings within a brood are not independent.

The model for ectoparasite infection intensity was fitted like the above, but with a generalized mixed model (GLMM) following a Poisson distribution with log-link function and the urban gradient as the key explanatory variable. Additional co-variables were brood size, sex, hatching rank, year and again brood ID as a random factor. Significance of explanatory terms in the GLMM was assessed using their partial (Type III) significance values (χ^2^ tests) implemented in the 'car' package (Fox and Weisberg 2011).

Best fitting models were chosen by model selection. We created candidate lists with all possible combinations of fixed effects (while keeping the main predictor of interest, urban gradient) and compared all candidate models via Akaike information criterion (AIC) for each response variable (Burnham and Anderson, 2002). The best models were chosen according to the lowest AIC value. To evaluate the proportion of variance explained by the models, pseudo *R*^2^ for LMMs and GLMMs was calculated following the method of Nakagawa & Schielzeth (2013) by using the function ‘r.squaredGLMM’ implemented in ‘MuMIn’ (Bartoń 2018). The complete models and most parsimonious (i.e. final models) are displayed in Appendix C.

All analyses were performed in R Version 3.4.3 (R Foundation 2018) using the following packages: ‘nlme’ (Pinheiro et al. 2018), ‘lme4’ (Bates et al. 2015), ‘MuMIn’ (Bartoń 2018), ‘AICcmodavg’ (Mazerolle 2017) and ‘lmerTest’ (Kuznetsova et al. 2017). Model validation was done by visual inspection of residual plots and tests for potential overdispersion. Results were visualised using ‘lattice’ (Sarkar 2008) and ‘ggplot2’ (Wickham 2009).

## Results

**Fig. 2:**
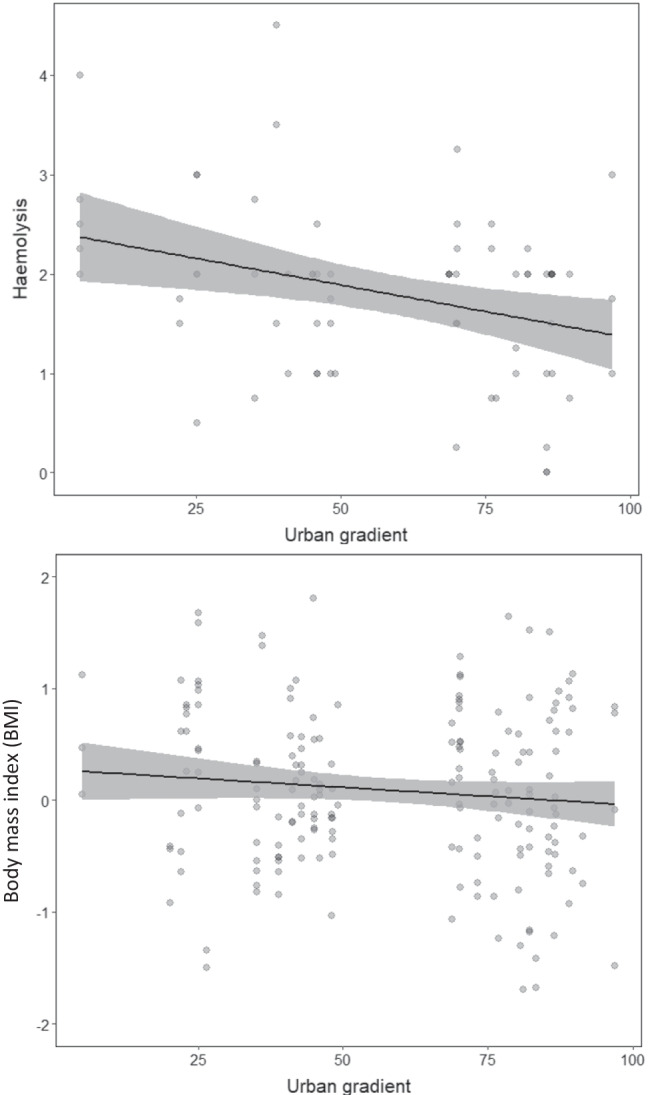
Effect of urban gradient on (top) haemolysis (*P* = 0.007, estimate =  − 0.34 ± SE 0.12), fitted with ectoparasite infection intensity as additional covariate. The model explains 30% of the variance in lysis; and on (bottom) body mass index (*P* = 0.019, estimate: − 0.45 ± SE 0.19), fitted with ectoparasite infection intensity and GSH:GSSG ratio as additional covariates. The model explains 71% of the variance in body mass index. Note that all continuous variables were scaled and centred. Figures are based on predicted values of LMMs, shaded grey areas represent 95% CIs overlaying the background scatter of raw data.

Kestrel nesting sites were recorded in areas between 5 and 95% of sealed surface (Fig. 1). There was no statistically significant relationship between the urban gradient and tGSH (*P* = 0.248, *R*^2^c = 0.21) or the ratio of GSH:GSSG (*P* = 0.997, *R*^2^c = 0.02, Table 1). Of the immune assays, lysis showed a significant negative relationship along the urban gradient (Estimate: − 0.34 ± SE 0.12, *P* = 0.007, *R*^2^c: 0.30, Table 2, Fig. 2), whereas neither haptoglobin concentration nor agglutination was related to urbanisation (haptoglobin, *P* = 0.945, *R*^2^c = 0.37; agglutination, *P* = 0.254, *R*^2^c = 0.43; Table 2).

**Table 1:** Results of LMMs used to examine the impacts of urban gradient and ectoparasite infection on oxidative stress parameters (total glutathione tGSH (*R*^2^c = 0.23) and GSH:GSSG ratio (*R*^2^c = 0.02)), fitted with additional covariates; ‘Brood ID’ was included as random factor; continuous variables were scaled and centred. *N* = 143 Eurasian kestrel *Falco tinnunculus* nestlings in Vienna, Austria. Significant predictors are displayed in bold.

Oxidative stress parameters	Estimate	Std. error	Sum Sq	Mean Sq	NumDF	DenDF	F value	Pr(> F)
tGSH								
Urban gradient	− 0.19	0.16	1.04	1.04	1	86.58	1.35	0.248
Ectoparasite Inf. Int			1.86	0.62	3	134.16	0.80	0.494
*Ectoparasite Inf. Int. (linear)*	− 0.28	0.31						0.368
*Ectoparasite Inf. Int. (quadratic)*	− 0.01	0.28						0.986
*Ectoparasite Inf. Int. (cubic)*	− 0.17	0.24						0.463
Plate ID ^±^			9.18	1.84	5	122.89	2.38	0.042
*Plate 2*	0.52	0.36						0.150
*Plate 3*	0.24	0.38						0.515
***Plate 4***	**0.99**	**0.31**						**0.001**
*Plate 5*	0.41	0.27						0.124
*Plate 6*	0.39	0.36						0.278
GSH:GSSG ratio								
Urban gradient	0.00	0.09	0.00	0.00	1	143.00	0.00	0.997
Ectoparasite Inf. Int			2.80	0.93	3	143.00	0.96	0.414
*Ectoparasite Inf. Int. (linear)*	0.36	0.23						0.122
*Ectoparasite Inf. Int. (quadratic)*	0.30	0.22						0.180
*Ectoparasite Inf. Int. (cubic)*	0.05	0.23						0.830

^±^Plate 1 was the reference category. *Ectoparasite Inf. Int.* ectoparasite infection intensity. *Std. error* standard error. *Sum Sq* sum of squares. *Mean Sq* mean squares. *Num DF* numerator degrees of freedom. *Den DF* denominator degrees of freedom.

**Table 2:** Results of LMMs used to examine the impacts of urban gradient on immune parameters (haptoglobin (*R*^2^c = 0.37), agglutination (*R*^2^c = 0.43) and lysis (*R*^2^c = 0.30)), fitted with further explanatory variables. To control for plasma colouration, we included 405 nm as covariate in haptoglobin models; ‘Brood ID’ was included as random factor in all models; continuous variables were scaled and centred. *N* = 69 Eurasian kestrel *Falco tinnunculus* nestlings in Vienna, Austria. Significant predictors are displayed in bold.

Immune parameters	Estimate	Std. error	Sum Sq	Mean Sq	NumDF	DenDF	F value	Pr(> F)
Haptoglobin								
Urban gradient	0.01	0.13	0.00	0.00	1	33.60	0.00	0.945
Ectoparasite Inf. Int			0.83	0.28	3	49.00	0.45	0.716
*Ectoparasite Inf. Int. (linear)*	0.09	0.31						0.786
*Ectoparasite Inf. Int. (quadratic)*	− 0.28	0.27						0.311
*Ectoparasite Inf. Int. (cubic)*	− 0.05	0.31						0.873
**Plasma colouration (405 nm)**	**1.75**	0.62	**4.93**	**4.93**	**1**	**61.86**	**8.08**	**0.006**
Agglutination								
Urban gradient	− 0.16	0.13	0.77	0.77	1	30.45	1.35	0.254
Ectoparasite Inf. Int			2.27	0.76	3	53.46	1.34	0.272
*Ectoparasite Inf. Int. (linear)*	0.24	0.32						0.459
*Ectoparasite Inf. Int. (quadratic)*	0.50	0.27						0.072
*Ectoparasite Inf. Int. (cubic)*	0.24	0.31						0.445
Lysis								
**Urban gradient**	− **0.34**	**0.12**	**5.77**	**5.77**	**1**	**33.70**	**8.26**	**0.007**
Ectoparasite Inf. Int			1.24	0.41	3	52.34	0.59	0.624
*Ectoparasite Inf. Int. (linear)*	− 0.38	0.30						0.216
*Ectoparasite Inf. Int. (quadratic)*	− 0.10	0.29						0.730
*Ectoparasite Inf. Int. (cubic)*	− 0.05	0.33						0.870

*Ectoparasite Inf. Int.* ectoparasite infection intensity*. Std. error* standard error. *Sum Sq* sum of squares*. Mean Sq* mean squares*. Num DF* numerator degrees of freedom*. Den DF* denominator degrees of freedom.

**Fig. 3:**
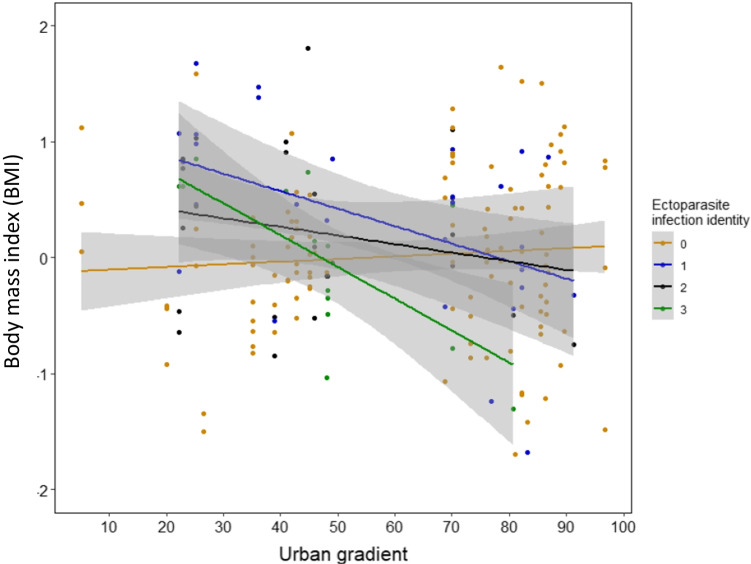
Effect of urban gradient on body mass index in interaction with ectoparasite infection intensity (*P* = 0.019, Estimate =  − 0.45 ± SE 0.19), fitted with and GSH:GSSG ratio as additional covariates. The model explains 71% of the variance in body mass index. Note that all continuous variables were scaled and centred. Figures are based on predicted values of LMMs, shaded grey areas represent 95% CIs overlaying the background scatter of raw data.

The body mass index decreased with increasing urbanisation levels (Estimate: − 0.45 ± SE 0.19, *P* = 0.019, *R*^2^c: 0.71, Table 3, Fig. 2). The body mass index was not significantly related to GSH:GSSG ratio nor to the interaction between the urbanisation and ectoparasite infection intensity; however, both terms still featured into the most parsimonious model (Table 3) and the interaction term was relevant to correctly interpret the urbanisation effect on the body mass index (Fig. 3). The interaction revealed that body condition of nestlings without ectoparasites did not vary along the urban gradient, while nestlings with an infection category of 1 or higher consistently had lower body condition in more urbanised areas (albeit statistically not significant; *P* = 0.181).

**Table 3:** Results of LMMs used to examine the impacts of urban gradient on body mass index, fitted with ectoparasite infection intensity and GSH:GSSG ratio as additional explanatory variable; ‘Brood ID’ was included as random factor; continuous variables were scaled and centred. *N* = 143 Eurasian kestrel *Falco tinnunculus* nestlings in Vienna, Austria (*R*^2^c = 0.71)

Body mass index	Estimate	Std. error	Sum Sq	Mean Sq	NumDF	DenDF	F value	Pr(> F)
**Urban gradient**	− **0.45**	**0.19**	1.94	1.94	1	112.38	5.64	**0.019**
Ectoparasite Inf. Int			1.43	0.48	3	119.59	1.39	0.249
*Ectoparasite Inf. Int. (linear)*	− 0.58	0.30						0.051
*Ectoparasite Inf. Int. (quadratic)*	− 0.29	0.21						0.160
*Ectoparasite Inf. Int. (cubic)*	0.03	0.17						0.876
GSH:GSSG ratio	0.03	0.06						0.647
UG: ectoparasite Inf. Int			1.70	0.57	3	119.80	1.65	0.181
*Ectoparasite Inf. Int. (linear)*	− 0.50	0.31						0.112
*Ectoparasite Inf. Int. (quadratic)*	− 0.04	0.23						0.844
*Ectoparasite Inf. Int. (cubic)*	− 0.22	0.19						0.253

*UG* urban gradient. *Ectoparasite Inf. Int.* ectoparasite infection intensity. *Std. error* standard error. *Sum Sq* sum of squares. *Mean Sq* mean squares. *Num DF* numerator degrees of freedom. *Den DF* denominator degrees of freedom.

**Fig. 4:**
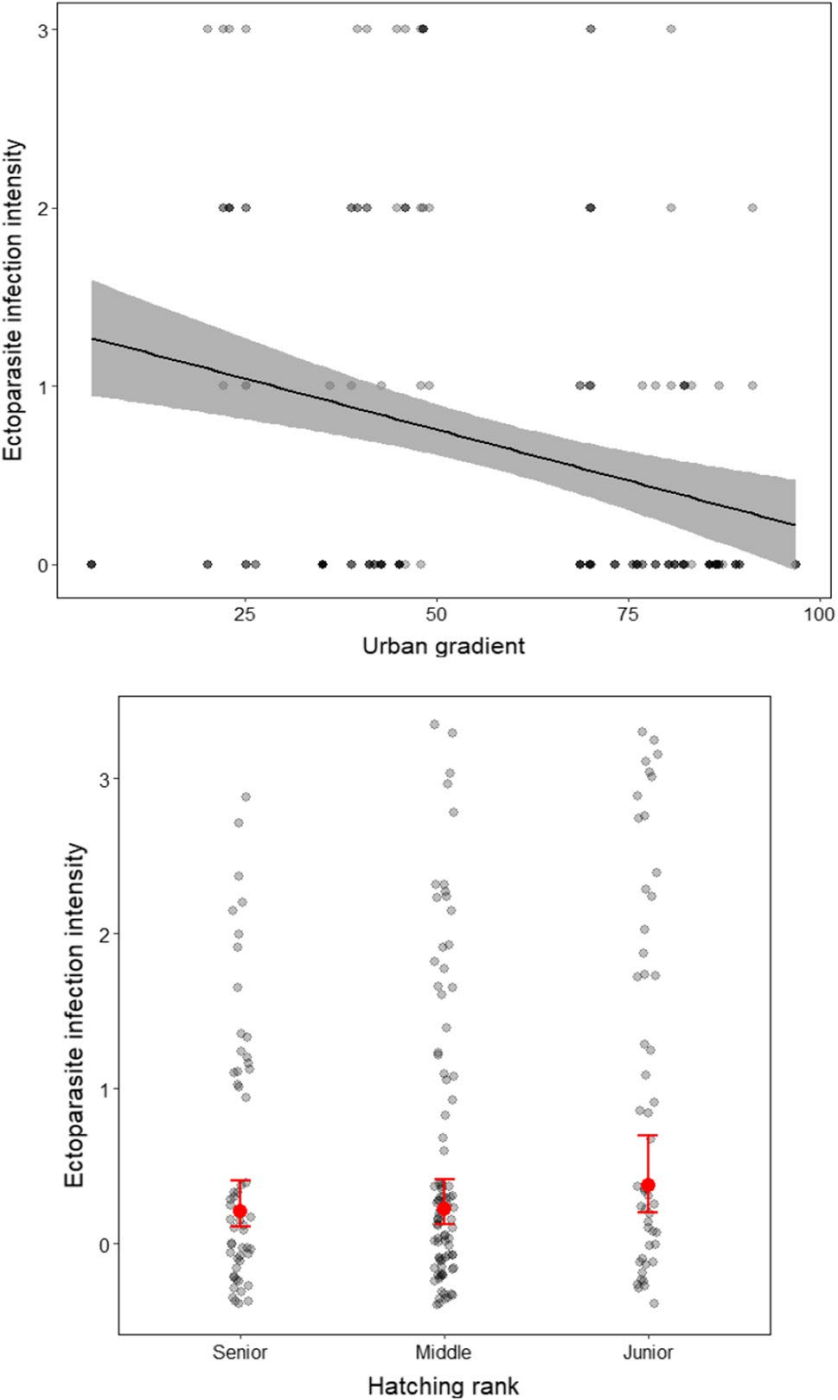
Ectoparasite infection intensity is influenced by (top) the urban gradient on (χ^2^ = 4.88, *P* = 0.027), and (bottom) hatching rank (χ^2^ = 8.33, *P* = 0.016), fitted with year as additional covariate (statistically not significant). The model explains 65% of the variance. Note that all continuous variables were scaled and centred. Figures are based on predicted values of GLMM, shaded grey areas represent 95% CIs, red dot and lines mean ± SEs overlaying the background scatter of raw data.

Ectoparasites (*Carnus hemapterus*) were found on 36.4% of all nestlings across both years, whereby infection intensity ranged from one to 40 individuals of *C. hemapterus* per individual (mean = 2.59 ± SE 5.93). Ectoparasite infection intensity was significantly lower in urban nestlings than in more rural individuals (Estimate: − 0.02 ± SE 0.009, *P* = 0.027, *R*^2^c = 0.65, Table 4, Fig. 4) and lower in senior siblings compared to their junior siblings (Fig. 4). In contrast to our predictions, ectoparasite burden did not influence any of the physiological parameters considered (tGSH, *P* = 0.494, *R*^2^c = 0.23, GSSG, *P* = 0.316, *R*^2^c = 0.04; GSH:GSSG ratio, *P* = 0.414, *R*^2^c = 0.02; see Table 1; haptoglobin, *P* = 0.716, *R*^2^c = 0.37; agglutination, *P* = 0.272, *R*^2^c = 0.43; lysis, *P* = 0.624, *R*^2^c = 0.3; see Table 2) or body mass index (*P* = 0.249, *R*^2^c = 0.71, see Table 3).

**Table 4:** Results of GLMMs used to examine the impacts of urban gradient on ectoparasite infection intensity, fitted with hatching rank and year as additional explanatory variable; ‘Brood ID’ was included as random factor. *N* = 195 Eurasian kestrel *Falco tinnunculus* nestlings in Vienna, Austria (*R*^2^c = 0.65). Significant predictors are displayed in bold.

Ectoparasite infection intensity	Estimate	Std. error	z-value	χ^2^	Pr(> z)
**Urban gradient**	− **0.02**	**0.009**	− **2.21**	**4.88**	**0.027**
**Hatching rank**				**8.33**	**0.016**
*Hatching rank. (linear) *	0.40	0.17	2.43		
*Hatching rank (quadratic) *	0.18	0.16	1.15		
Year (2017) ^±^	− 0.83	0.47	− 1.78	3.14	0.076
*Intercept*	*0.23*	*0.58*	*0.39*	*0.16*	*0.693*

Std. error standard error ± year 2015 was used as reference category.

## Discussion

In the present study, we used a physiological biomarker approach—combining two systems, oxidative stress and innate immune function—to investigate how an avian predator responds to urbanisation and its associated challenges. Nestlings of the Eurasian kestrel had lower lysis (complement activity) and lower body mass index in areas with more sealed surface, providing an indication that urban kestrels might suffer from some hidden costs in terms of reduced immune capacities and physical condition. On the other hand, ectoparasite infection intensity of nestlings was overall lower in more urbanised areas. There was no correlation between physiological parameters measured in our study and ectoparasite burden and several other physiological parameters showed no correlation with the urban gradient.

As predicted, we found a negative correlation between lysis (complement activity) and the urban gradient. Lysis has previously been found to be positively correlated with survival in wild birds (Hegemann et al. 2017); thus, a higher lysis capacity should be beneficial when parasitic prevalence is high (Bradley & Altizer 2007). This is in accordance with our result whereby non-urban nestlings showed higher lysis and also higher ectoparasite infection intensity with *C. hemipterus.* In altricial birds, a significant part of innate immune function develops during the nestling phase (Palacios et al. 2009; Killpack et al. 2013; Aastrup & Hegemann 2021). Thus, as an alternative explanation of our results of higher lysis in kestrel nestlings in the less urbanised areas could indicate an earlier and/or stronger development of the innate immune system compared with nestlings from more urban areas. Perhaps the weaker and/or delayed onset in urban kestrel nestlings is linked to an altered diet in urban surroundings (Sumasgutner et al. 2014a, b, c), but it could also reflect an overall lower abundance of pathogens.

In contrast to the lysis capacity, none of the other physiological parameters measured were affected by urbanisation. For example, urbanisation was not associated with our measurements of the antioxidant system (tGSH and GSH:GSSG ratio), natural antibodies (agglutination) or inflammation levels (haptoglobin).

We calculated the urban gradient as a percentage of sealed surface; given the large amount of studies showing a correlation between urbanisation and urban factors (Alberti et al. 2001; McDonnell and MacGregor-Fors, 2016; McDonnell and Hahs, 2008), we tentatively assume that our urbanisation metric correlates with these other urban stressors but we have unfortunately no data from our own system to support this. In addition, it is possible that these stressors are differently pronounced at the micro-habitat scale, i.e. depending on nest type. In comparison with open nests, cavities are less exposed to the external environment (Kreiderits et al. 2016; see also results on urban peregrine falcons in Sumasgutner et al. 2020); thus, they might better shelter against light, noise and chemical pollution. Because nest type is largely confounded with location along the urban gradient, with more cavities in more urbanised areas and more open nests in planters and trees in less urbanised areas, it is unfortunately not possible to tease apart these two factors. Additionally, the different urban stressors might influence physiological parameters differently, which could only be addressed with an experimental study design. Furthermore, these urban stressors hardly act in separation, and the combined exposure to multiple urban stressors may either show additive or synergistic effects (see also Isaksson 2015). For example, there may be negative effects on physiological health exerted by chemical pollution (Isaksson 2010; Koivula & Eeva 2010); the inhalation of NOx causes a series of redox reactions in the airways and ultimately triggers both antioxidant and inflammatory responses (Last et al. 1994). Rising levels of oxidative stress and inflammation have also been demonstrated in mice exposed to noise (Münzel et al. 2017). Noise pollution impairs several behavioural patterns of wild animals (Barber et al. 2009; Kight & Swaddle 2011) and may also affect GSH levels due to its dual function as antioxidant and neurotransmitter/modulator (Janáky et al. 1999; Hovatta et al. 2005; Yamane et al. 2007). The exposure to artificial light at night can change reproductive, feeding and sleeping behaviour (Kempenaers et al. 2010; Le Tallec et al. 2013) often resulting in an increased activity (e.g. extended foraging time or disturbed sleep), which further raises the metabolic demand and ultimately affects oxidative stress (Metcalfe and Alonso-Alvarez 2010; Selman et al. 2008). Artificial night light can also trigger suppression of cell-mediated and humoral immune functions in birds and mammals (Moore & Siopes 2000; Bedrosian et al. 2011). Yet, the present data reveal no effect on the measured GSH, the GSG:GSSG ratio, haptoglobin nor agglutination.

The body mass index of kestrel nestlings decreased with increasing urbanisation. An increase of soil sealing is linked to lower accessibility of diurnal rodents as suitable prey (Sumasgutner et al., 2014a; Mitter et al. 2015) and typically results in a pronounced shift in diet composition towards avian prey (Kübler et al. 2005; Sumasgutner et al., 2014a, b). Passerines are poorer in nutritional value compared to voles (Goodwin 1980; Kirkwood 1991) and kestrels are not necessarily capable of catching agile birds to sufficiently provision offspring with prey items (Fargallo et al. 2020). The resulting malnutrition typically affects the junior/youngest siblings, which additionally suffer from higher ectoparasite burden (‘tasty chick hypothesis’; Roulin et al. 2003; Sumasgutner et al., 2014c). Both mechanisms reduce brood size after hatching (Sumasgutner et al., 2014b). The lack of suitable prey items has cascade effects on essential dietary antioxidants such as carotenoids (Isaksson and Andersson 2007; Sumasgutner et al. 2018), which can be further enhanced at higher trophic levels (Drouillard et al. 2001; Henny et al. 2003). Thus, the most likely explanation for our lower body mass index in urban kestrel nestlings is the lack of suitable prey. This finding is in accordance with other urban-dwelling avian species (see Liker et al. 2008; Meillère et al. 2015; Herrera-Dueñas, 2018; Isaksson et al., 2005; Chamberlain et al., 2009). However, a recent urban raptor study showed that offspring with lower body mass indices also had higher local apparent survival rates (Nebel et al. 2021)—unfortunately we do not have data on post-fledging survival in our population from which to infer potential long-term fitness consequences.

Wildlife parasites tend to be less diverse in urban areas, yet parasite transmission can occur more rapidly because of higher avian density, avian aggregations at supplementary feeding stations and/or the close proximity between wild animals, livestock, pets and humans (Bradley & Altizer, 2007). In this study, we found fewer ectoparasites per nest with increasing urbanisation. Literature on parasitaemia along urban–rural gradients is rare but shows both negative and positive trends. For example, the risk of haemoparasite infection decreased with increasing urbanization (Fokidis et al. 2008; Bailly et al. 2016; Suri et al. 2017), while prevalence of coccidia, poxvirus and trichomoniasis increased with urbanization (Giraudeau et al. 2014; Mannan et al. 2008). Avian malaria (*Plasmodium* sp.) has been shown to trigger expression of antioxidant and immune genes (Videvall et al. 2015) and increase oxidative damage measured as reactive oxygen metabolites (Isaksson et al. 2013). To date, few studies have investigated the physiological signals of stress caused by ectoparasites. Short-term expression of antioxidants and immune response, on the other hand, has been shown to increase during peak parasitaemia in birds infected with malaria (Videvall et al. 2015). Few studies consider physiological signals of stress caused by ectoparasites (but see Dudaniec et al. 2006; Fessl et al. 2006; Sun et al. 2020), in which bites create small wounds with oral secretion inserted into the host’s skin tissue that may trigger an inflammatory response (Baron and Weintraub 1987; Owen et al. 2010). From such wounds, various immune cells generate reactive oxygen species (Halliwell & Gutteridge 2002; Sorci & Faivre 2009; Costantini & Møller 2009) and excessive reactive oxygen species production may also affect host tissues, resulting in oxidative stress (Sorci & Faivre 2009). We found no significant effects of *C. hemapterus* intensity on physiological stress parameters examined in this study. Our data are a snapshot of the nestling phase, whereby nestlings were measured and sampled at age 12 to 16 days. The peak infection with *C. hemapterus* in Common Starlings (*Sturnus vulgaris*, Liker et al. 2001) and American kestrels (*Falco sparverius*, Lesko and Smallwood 2012) occurred earlier in the nestling period, while barn owls did not show a significant relationship between nestling age and *C. hemapterus* infection intensity (Roulin et al. 2007). We do not know when *C. hemapterus* peaks in European kestrels (only that infection is lower in the age range 16–25 than in the age range 6–15 days; Sumasgutner et al., 2014c), but if the peak in this species is also earlier, then this may have contributed to the lack of any significant relationship with physiological parameters. In any case, early mortality is not visible in our data set especially when considering that clutch sizes are similar along the urban gradient but the number of fledglings is significantly smaller in the most urban parts (Sumasgutner et al., 2014a). While it is to be expected that this bias affects all nests in our study system equally, it might still skew the results if junior siblings in highly urbanised areas are indeed dying early while also having the highest ectoparasite burden as suggested by the ‘tasty chick hypothesis’ (Roulin et al. 2003). The result would be an overall healthier but smaller brood. However, the tasty chick hypothesis is not applicable to all avian systems. For example, Valera et al. 2004 found that *C. hemapterus* tend to aggregate on the larger hatchling in European bee-eaters *Merops apiaster*, whereby ectoparasites were not associated with level of immunocompetence; without repeated measures throughout the nestling period, we cannot identify the peak of infection intensity. Another reason might be that our categorical estimation of ectoparasites does not provide sufficient resolution to capture the full variation of parasite levels. For future studies, we suggest repeated sampling including the early (and late) nestling stage as well as actual parasite counts rather than categorical estimates.

Ectoparasites can cause both short- or long-term fitness costs (Fitze et al 2004). Thus, we predicted that *C. hemapterus* and the body mass index of the kestrel nestlings would be negatively correlated, yet we did not find such a pattern. Possibly this is due to the large effect of urbanisation on body mass index, which goes in the opposite direction to parasite intensity. Thus, lack of suitable and abundant prey has a larger influence on body mass index than parasite load. Furthermore, urban environments are generally warmer than rural environments due to the so-called urban heat island effect (Oke 1982; Solecki et al 2005; Morabito et al 2016). Experimental manipulation of in-nest temperature in blue tit *Cyanistes caeruleus* nest boxes resulted in fewer mites and blowfly pupae, and a trend for fewer fleas (Castaño-Vázquez et al. 2018). It is possible that our finding of fewer *C. hemapterus* in urban areas could be the consequence of higher temperatures along with more cavity nests in urban areas, which remains to be investigated in more detail. More research into the causes and consequences of ectoparasitism is needed to more fully understand possible interaction effects along urban–rural habitat areas.

## Conclusion

Living in urban environments can affect pathogen pressure, immune defences and oxidative stress (Audet et al. 2016; Bailly et al. 2016; Capilla-Lasheras et al. 2017) and despite some non-significant results, our study provides support that raptors that attain high breeding densities in urban environments can experience potentially negative health impacts. Complement activity (lysis) and the body mass index were both lower in areas with more sealed surface area, despite an overall lower ectoparasite infection intensity with *C. hemipterus*. Due to the complexity of the antioxidant and immune systems and the strong connection of impervious surface and urban stressors, further studies on a broader scale, especially over several breeding seasons with greater sample sizes and including adults as well as nestlings, will be needed to disentangle the detailed underlying mechanisms.

## Supplementary Information

Below is the link to the electronic supplementary material.

Supplementary file (DOCX 218 KB)

## Data Availability

The data underlying this study are available as supplementary electronic material. The study was performed under licence of the Environmental Protection Bureau of Vienna (MA22/1263/2010/3), the Ministry for Science and Research BM.WF (BMWF –66.006/0010-II/3b/2011 and 0021II/3b/2013, 2015, 2017) and was approved by the ethics committee of the University of Veterinary Medicine, Vienna (TGV, BGBI.Nr.501/1989 i.d.F. BGBI. I Nr.162/2005). All data were acquired strictly following current Austrian and EU law as well as the guidelines for treatment of animals in behavioural research and teaching (ASAB2018).
