# Supplementary material for: Reduced ectoparasite load, body mass and blood haemolysis in Eurasian kestrels (Falco tinnunculus) along an urban–rural gradient
Source: Naturwissenschaften. 2021 Sep 7;108(5):42. doi: 10.1007/s00114-021-01745-x (PMC8423637; doi:10.1007/s00114-021-01745-x)
Supplement: Supplementary file 1 — Supplementary file (DOCX 218 KB) [file 114_2021_1745_MOESM1_ESM.pdf]

SUPPLEMENTARY MATERIAL

Reduced ectoparasite load, body mass and blood haemolysis in Eurasian kestrels (*Falco tinnunculus*) along an urban-rural gradient

Laura Wemer, Arne Hegemann, Caroline Isaksson, Carina Nebel, Sonia Kleindorfer, Anita Gamauf, Marius Adrion, Petra Sumasgutner

Appendix A - Laboratory protocol of Oxidative Stress Assays

Measuring Total and Oxidised Glutathione in Whole Blood
(established by Caroline Isaksson, June 2009)

To measure total (tGSH) and oxidised glutathione (GSSG) we followed the method of Baker et al. (1990), adapted to a microplate reader by Vandeputte et al. (1994; see also Stephensen et al., 2002 and Isaksson 2013) and for use with an injector.

We used 4 µl of whole blood for the glutathione measurement. First, blood cells were lysed using 16 µl 5% sulfosalicylic acid (SSA) and centrifuged at 10,000 rpm for 10 min at 4 °C. 10 µl of the supernatant was transferred into a new tube and diluted with 200 µl GSH buffer (which contained 143 mM NaH_2PO_4, 6.3 mM EDTA, pH 7.4). 100 µl of the dilution were transferred to another tube which contained 5 µl of 4-vinylpyridine (4-VNP). 4-VNP derivatises GSH and therefore prevents it from inferring with the GSSG measurement. Samples for the GSSG analysis were incubated at room temperature for an hour and centrifuged before analysis at 12,000 rpm for 5 min at 4 °C. Standards, samples, and reagent stocks were kept on ice until transfer to a 96-well microtitre plate. For GSH standards, a 10 mM GSH stock solution was diluted with 0.5% SSA to the concentrations 1.875, 3.12, 6.25, 12.5, 25, 50 and 100 µM. GSSG standards were prepared by diluting a 5 mM stock solution to the concentrations 0.1875, 0.3125, 0.625, 1.25, 2.5, 5 and 10 µM. All samples and standard curves were run in duplicates with each plate containing one standard curve. 20 µl of sample or standard were added to a well. As the assay is very time sensitive from the moment NADPH (see below) is added to the well, only half a plate at a time was filled with samples.

200 µl of the working solution (4 ml DTNB, 29.2 GSH-buffer and 1 ml 0.34 units/well GR for GSH or 1.2 ml 0.17 units/well GR for GSSG, amount sufficient for 2 plates) were added to the wells. Afterwards, the plate was immediately placed in a FLUOstar Omega microplate reader (BMG Lab-tech). 34 µl NADPH was added by an injector by the plate reader before the start of measurements. Change in absorptance was observed at room temperature at 412 nm every 30 s for 5 minutes and compared to a simultaneously obtained standard curve from GSH or GSSG with known concentration.

All chemicals were mixed up in the following way:

GSH-Buffer: 143 mM $NaH_2PO_4$and 6.3 mM EDTA, pH level 7.4 (2.3452 g EDTA + 19.732 g NaH2PO4; and approx. 800 ml ddH2O (= purified H2O))

Kept at room temperature:

5% SSA: 50 mg in 1.5 ml tube (for add 1 ml GSH-buffer shortly before further processing)

0.5% SSA: 50 mg in 15 ml tube (for add 10 ml GSH-buffer shortly before further processing)

DTNB: 20.6 mg in 15 ml tube (for add 5.2 ml GSH-buffer shortly before further processing)

Kept at -20°C:

NADPH: 12.5 mg in 15 ml tube (for add 7.5 ml GSH-buffer shortly before further processing)

4-VNP (GSSG only): 100 µl 4-VNP in 1.5 ml tube (for add 400 µl EtOH 100%)

Kept at – 80°C:

GSH standard 10 mM: 30.7 mg in 15 ml tube (for add 10 ml GSH-buffer shortly before further processing; 50 µl aliquots were transferred into 0.5 ml tubes and frozen a.s.a.p.)

GSSG standard 5 mM:

32.8 mg in 15 ml tube (for add 10 ml GSH-buffer shortly before further processing; 50 µl aliquots were transferred into 0.5 ml tubes and frozen a.s.a.p.). Aliquots of GSH and GSSG were taken from -80°C to -20°C when assays were started.

Glutathione Reductase (GR):

For GSHt assay: for 1 ml of GR (= 0.068 U/µl) we calculated the following (V_0): $V_0 = \frac{0.068 \text{ U per } \mu\text{l} \times 1000\ \mu\text{l}}{[\text{GR}]\text{per } \mu\text{l batch}}$, put it in a 1.5 ml tube, centrifuged it at 13000 rpm for 3 minutes, removed the supernatant, refilled it up to 1 ml with GSH-buffer and mixed carefully.

For the GSSG assay, 0.3 ml were transferred into a new 1.5 ml tube and diluted 1:1 with GSH-buffer.

Nunclon 96-well MicroWell plates were purchased from VWR Sweden

Appendix B - Laboratory protocol of Immune Assays

Haptoglobin

We quantified haptoglobin concentrations (mg mL^{-1}) in plasma samples using a commercially available colorimetric assay kit (TP801; Tri-Delta Diagnostics, NJ, USA). This functional assay calorimetrically quantifies the heme-binding capacity of plasma. We followed the "manual method" instructions provided by the kit manufacturer with a few minor modifications following Matson et al. (2012). We measured absorbance at two wavelengths (405 and 630 nm) prior to the addition of the final reagent that initiated the colour-change reaction. We used the pre-scan at the normal assay wavelength of 630 nm to correct for differences in plasma redness by subtracting pre-scan absorbance values from final absorbance values. We used the 450 nm pre-scan to statistically correct for differences in plasma sample redness, an indication of haemolysis, which can affect the assay (Matson et al. 2012).

Hemolysis-hemagglutination

We quantified complement activity and natural antibody titres following the method described by Matson et al. (2005). In brief, red blood cells (RBC) from rabbits (Envigo, United Kingdom) were incubated in serially diluted plasma samples. Agglutination and lysis were recorded as titres (−log2 of the last plasma dilution that shows each reaction). We used assay plate images taken 20 min after incubation to score agglutination and images made 90 min after incubation to score lysis. Images were randomised and scored at least twice by AH who was blind with respect to bird ID.

Appendix C - Response variables and co-variates that were additionally explored to the key explanatory variable ('urban gradient', not shown) in this study on urban Eurasian kestrels in Vienna, Austria. Co-variates used in the final LMMs and GLMM (for ectoparasite infection intensity as response variable) were chosen by model selection (AIC). In all models 'brood ID' was added as a random factor.

**Response variable**	**Co-Variates (initially fitted)**	**Co-Variates (of most parsimonious model after AIC-based model selection)**	**Sample size (N)**
tGSH	ectoparasite infection, brood size, sex, hatching rank, plate ID	ectoparasite infection, plate ID	143
GSH:GSSG ratio		ectoparasite infection	
Haptoglobin	ectoparasite infection, brood size, sex, hatching rank, plasma colouration	ectoparasite infection, plasma colouration	69
Haemagglutination	ectoparasite infection, brood size, sex, hatching rank	ectoparasite infection	
Haemolysis			
body mass index	ectoparasite infection, GSH:GSSG ratio, brood size, hatching rank	ectoparasite infection intensity, GSH:GSSG ratio	143
Ectoparasite infection	brood size, sex, hatching rank, year	hatching rank, year	195

REFERENCES

Isaksson C, Sheldon BC, Uller T (2011) The Challenges of Integrating Oxidative Stress into Life-history Biology. BioScience 61: 194–202.

Matson KD, Ricklefs RE, Klasing KC (2005) A hemolysis–hemagglutination assay for characterizing constitutive innate humoral immunity in wild and domestic birds. - Developmental & Comparative Immunology 29: 275–286.

Matson KD, Horrocks NPC, Versteegh MA, Tieleman BI (2012) Baseline haptoglobin concentrations are repeatable and predictive of certain aspects of a subsequent experimentally-induced inflammatory response. Comparative Biochemistry and Physiology. A: 162: 7–15.

Stephensen E, Sturve J, Forlin L (2002) Effects of redox cycling compounds on glutathione content and activity of glutathione related enzymes in rainbow trout liver. Comparative Biochemistry and Physiology. C: Toxicology and Pharmacology 133: 435–442.

Vitousek PM, Mooney HA, Lubchenco J, Melillo JM (1997) Human Domination of Earth's Ecosystems. Science 277: 494–499.
